# Cytokeratin 20 positive circulating tumor cells are a marker for response after neoadjuvant chemoradiation but not for prognosis in patients with rectal cancer

**DOI:** 10.1186/s12885-015-1989-z

**Published:** 2015-12-16

**Authors:** Sebastian Hinz, Christian Röder, Jürgen Tepel, Alexander Hendricks, Clemens Schafmayer, Thomas Becker, Holger Kalthoff

**Affiliations:** Department of General and Thoracic Surgery, University Hospital Schleswig-Holstein, Campus Kiel, Arnold-Heller Str. 7, 24105 Kiel, Germany; Division Molecular Oncology, Institute for Experimental Cancer Research, Cancer Center North, University Hospital Schleswig-Holstein, Campus Kiel, Arnold-Heller Str. 7, 24105 Kiel, Germany; Clinic for General, Thoracic and Visceral Surgery, Klinikum Osnabrück, Am Finkenhügel 1, 49076 Osnabrück, Germany

**Keywords:** Rectal cancer therapy, Circulating tumor cells, CK20 RT-PCR, Response to chemoradiation

## Abstract

**Background:**

Several studies have shown, that circulating tumor cells (CTC) have a negative prognostic value in colorectal cancer patients. Aim of this study was to evaluate the role of CTC in specifically rectal cancer patients regarding the influence on overall survival and to elucidate the impact of CTC in predicting response after chemoradiation (RCTX).

**Methods:**

In this prospective monocentric study 267 patients with rectal cancer were included. Patients with locally advanced tumors were treated with RCTX followed by surgery. The primary endpoints were: Evaluation of CTC at the time of surgery and correlation with main tumor characteristics, response to neoadjuvant RCTX and overall survival (OS). CTC were detected in the blood using CK20 RT-PCR.

**Results:**

Sixty-three patients were treated with neoadjuvant RCTX. In 46.8 % of the patients receiving neoadjuvant RCTX CTC were detected, which was significantly higher than in the group without RCTX (*p* = 0.002). Histopathologic regression after RCTX was evident in 27.8 % of the patients. In the subgroup of responders after RCTX we found CTC at a significantly lower rate than in non-responders (*p* = 0.03). No significant association was found between CTC detection and tumor characteristics and OS. The OS was significantly improved for responders compared to non-responders (*p* = 0.007).

**Conclusions:**

Responders after neoadjuvant RCTX had a lower incidence of CTC compared to non-responders, which might be a result of effective systemic and local treatment prior to surgery. Interestingly, detection of CTC did not correlate with tumor stage and OS, which is in contrast to previous reports of patients with colon cancer.

**Electronic supplementary material:**

The online version of this article (doi:10.1186/s12885-015-1989-z) contains supplementary material, which is available to authorized users.

## Background

Despite increasing efforts being made in the past with prevention programs and strategies to improve therapeutic efficiency with neoadjuvant chemoradiation, adenocarcinoma of the rectum is still one of the most common malignancies in the western world. A five-year relative survival rate of nearly 60 % in Europe in the last years [1] implies that several patients develop disease recurrence, primarily with liver and lung metastases.

Recently, a variety of molecular biomarkers and high-risk gene signatures have been introduced that may provide further information regarding prognosis and risk stratification to neoadjuvant treatment or adjuvant therapy of patients with colorectal cancer (CRC) [2, 3]. None of these parameters have been implemented in routine clinical practice with the exception of mutational *KRAS* status in patients with advanced CRC [4]. The existence of circulating tumor cells (CTC) has been known for years. With the improvement of molecular detection technologies of CTC during the last years, making tests easier and more reliable, the clinical perception has been underlined. Recently the identification of single tumor cells in the blood or bone marrow has been proposed as a prognostic biomarker for colorectal cancer and other malignancies [5]. It has been shown for colorectal cancer that patients with circulating tumor cells in the blood have a shorter overall survival (OS) [6]. This was supported by a recent meta-analysis that could reveal CTC as a significant negative prognostic factor in a pooled analysis [7]. We were also able to show that disseminated tumor cells (DTC) in the bone marrow negatively influence OS in patients after complete resection of colorectal liver metastasis [8]. Furthermore, liver resection and radiofrequency ablation for liver metastases can considerably change the level of CTC in the blood [9]. On the other hand, test methods and markers for CTC and DTC in CRC patients are still not standardized and prospective, multicenter trials with large patient numbers are needed.

Response to preoperative chemoradiation can sometimes even achieve a complete pathological remission, which is an important prognostic factor for locally advanced rectal cancer. During the last years, several studies have described markers and gene expression profiles to predict response to neoadjuvant chemoradiation [10, 11]. Despite some encouraging results in defining markers/ gene profiles, there is still controversy between different studies.

A small series of 26 patients with rectal cancer undergoing neoadjuvant chemoradiation was able to show that responders to RCTX have a higher rate of CTC before initiation of RCTX compared to non-responders, and that RCTX induces a significant decrease in the detection rate of CTC in responders [12]. Thus, we designed this study to further evaluate the prognostic value of CTC and to elucidate the impact of CTC in predicting response after RCTX. To our knowledge, this is the largest series of patients evaluating the impact of CTC in rectal cancer patients.

## Methods

### Patients

A total of 267 patients with histopathologically confirmed rectal cancer who underwent surgery in our department were included. The study was approved by the local ethics committee (Christian-Albrechts-University Kiel, Faculty of Medicine; Az. 99/110) and all patients gave written informed consent. In addition to endoscopic and histological evaluation, all patients were staged using computed tomography of the abdomen, chest X-ray and rectal endosonography or a pelvic magnetic resonance imaging (MRI). Classification of the pathological cancer staging and grade was performed at the Department of Pathology, University Hospital Schleswig-Holstein, Campus Kiel. Patient’s overall survival was the main endpoint result of our study and was further determined as the number of months between the date of surgery and the date of death or date of the last follow-up of patients. Clinical follow-up was performed in cooperation with general practitioners. Data were fed into a web-based research database, developed at our department (http://www.prowebdb.de). Patients with a locally advanced disease (cT3/T4 or cN1) were scheduled for neoadjuvant chemoradiation with 50.4 Gy and two cycles of chemotherapy with 5-fluorouracil (5-FU) followed by 4 cycles of chemotherapy with 5-FU after surgery (according to [13]). In some cases this workflow deviated according to the consensus meeting of the interdisciplinary tumor board. All patients that received neoadjuvant chemoradiation were restaged with rectal endosonography to determine the extent of tumor response directly before the operation.

### Sample collection, isolation of RNA and RT-PCR

From each patient blood samples (10 ml) were obtained promptly ahead of skin incision from a central line. Mononuclear cells (MNC) were extracted using density centrifugation through Ficoll-Hypaque. As described previously, RNA was extracted from MNC fractions and CK20-RT-PCR was performed [14]. Samples were tested twice, in the case of two inconsistent results a third analysis was performed. Two positive PCR results were needed to judge a final positive test result.

### Statistical analysis

Univariate Kaplan-Meier survival analysis was performed to estimate overall survival (OS) in dependence on the CK20-status in the blood. The detection rate of CTC and correlation with clinicopathologic parameters was analyzed with the χ^2^ test after crosstab analysis. OS was summarized using the Kaplan-Meier method. The differences in the survival curves of the subgroups were assessed by the log-rank test. Independence of categorical variables was tested by Pearson’s χ^2^ test after crosstab analysis. All reported *p*-values are two-sided and differences were judged significant if *p* was 0.05 or less. Calculations and tests were performed with SPSS 22.0 (SPSS Inc., Chicago, IL).

## Results

### Clinical characteristics

Our study population consisted of 267 patients with rectal cancer. The mean age at the time of surgery was 64.4 years (range 30–90 years). The clinical and histological parameters are summarized in Table 1. From all patients, 79/ 267 (29.6 %) underwent neoadjuvant chemoradiation. Sixty-three patients received adjuvant chemoradiation, resulting in a total of 142 patients (53.2 %) receiving chemoradiation. In patients with tumor localization in the middle and lower 1/3 of the rectum 41/ 109 (37.6 %) and 27/ 63 (42.8 %) patients, respectively, received neoadjuvant chemoradiation, whereas 11/ 94 patients showing a tumor in the upper 1/3 of the rectum were scheduled for preoperative chemoradiation (*p* < 0.001). Two patients had a complete remission after chemoradiation without any detectable tumor cells in the resected specimen.

**Table 1:** Patient characteristics

Characteristics	Category	Number	Percent
Sex	Male	141	52.8
	Female	126	47.2
Age	Years^a^	64.4 (30–90)	
UICC stage	I	82	30.7
	II	72	27
	III	85	31.8
	IV	28	10.5
Neoadjuvant RCTX	Yes	79	29.6
	No	188	70.4
Adjuvant RCTX	Yes	63	23.5
	No	204	76.5
Responder RCTX	Yes	22	27.8
	No	57	72.2
Tumor localisation	Upper 1/3	94	35.2
	Middle 1/3	109	40.8
	Lower 1/3	63	23.6
Blood CK20	Negative	173	64.8
	Positive	80	30
	Not done	14	5.2

^a^Data are expressed as means (range)

### Correlation of tumor stage and overall survival

According to the UICC classification patients were divided into groups with different tumor stages. The overall survival was highly related to the tumor stage with five-year survival rates of 86 % for stage 1, 71 % for stage 2, 62 % for stage 3 and only 21 % for stage 4 (*p* < 0.001, log rank test). The extent of lymph node metastases did also significantly influence overall survival. The five-year survival rate varies from 76 % for patients with pN0 stage, 73 % for pN1 stage and 40 % for patients with pN2 stage (*p* < 0.001, log rank test).

### Detection of CK20-positive circulating tumor cells in the blood

**Fig. 1:**
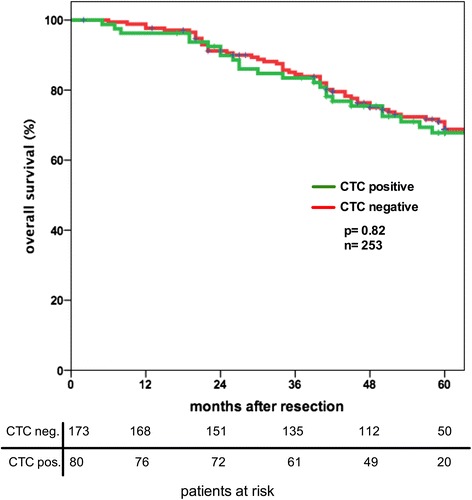
Detection of CTC in rectal cancer patients is not a prognostic marker for overall survival

Blood samples of 253 patients with rectal carcinoma were analyzed with CK20 RT-PCR prior to surgery. The sensitivity and specificity of the CK20 RT-PCR has been described earlier by our group [14, 15]. A sample result of the CK20 RT-PCR with 16 patients tested including two positive controls is shown in Additional file 1: Figure S1. In 31.8 % (80/253) of all patients circulating tumor cells were detected in the peripheral blood. In 46.8 % (37/79) of patients with locally advanced rectal cancer receiving neoadjuvant chemoradiation CTC were detected compared to only 24.7 % (43/174) in the group without chemoradiation (*p* = 0.002 χ^2^-test, Table 2). This reflects that patients with locally advanced tumors are more likely to have CTCs, although the univariate analysis of tumor localization, nodal status and tumor stage did not show a significant correlation with the presence of circulating tumor cells (Table 2). The overall survival of patients with circulating tumor cells in the blood was not significantly different from patients without these cells (Fig. 1). Also subgroup analysis including only patients without RCTX detection of CTC was not a prognostic marker. In our study population we were not able to show a significant difference in the overall survival of patients with UICC stage I + II disease with or without CTC (data not shown). No statistically significant differences in local recurrence rate and development of metastases were apparent between patients with or without CTC detection.

**Table 2:** Detection of CK20 positive tumor cells (CTC) in the blood of patients with rectal cancer

Variable	Category	Rate of CK20 positive CTC	χ^2^-test (p-values)
Neoadjuvant RCTX	Yes	46.8 % (37/79)	*p* = 0.002
	No	24.7 % (43/174)	
Tumor localisation	Upper 1/3	31 % (27/87)	*p* = 0.42
	Middle 1/3	27.6 % (29/105)	
	Lower 1/3	37 % (23/61)	
pN- status	pN+	30 % (31/103)	*p* = 0.6
	pN-	32.6 % (49/150)	
UICC stage	I	30.6 % (23/75)	*p* = 0.99
	II	32.3 % (23/71)	
	III	31.3 % (25/80)	
	IV	33.3 % (9/27)

### Influence of neoadjuvant chemoradiation on tumor response and circulating tumor cells

**Fig. 2:**
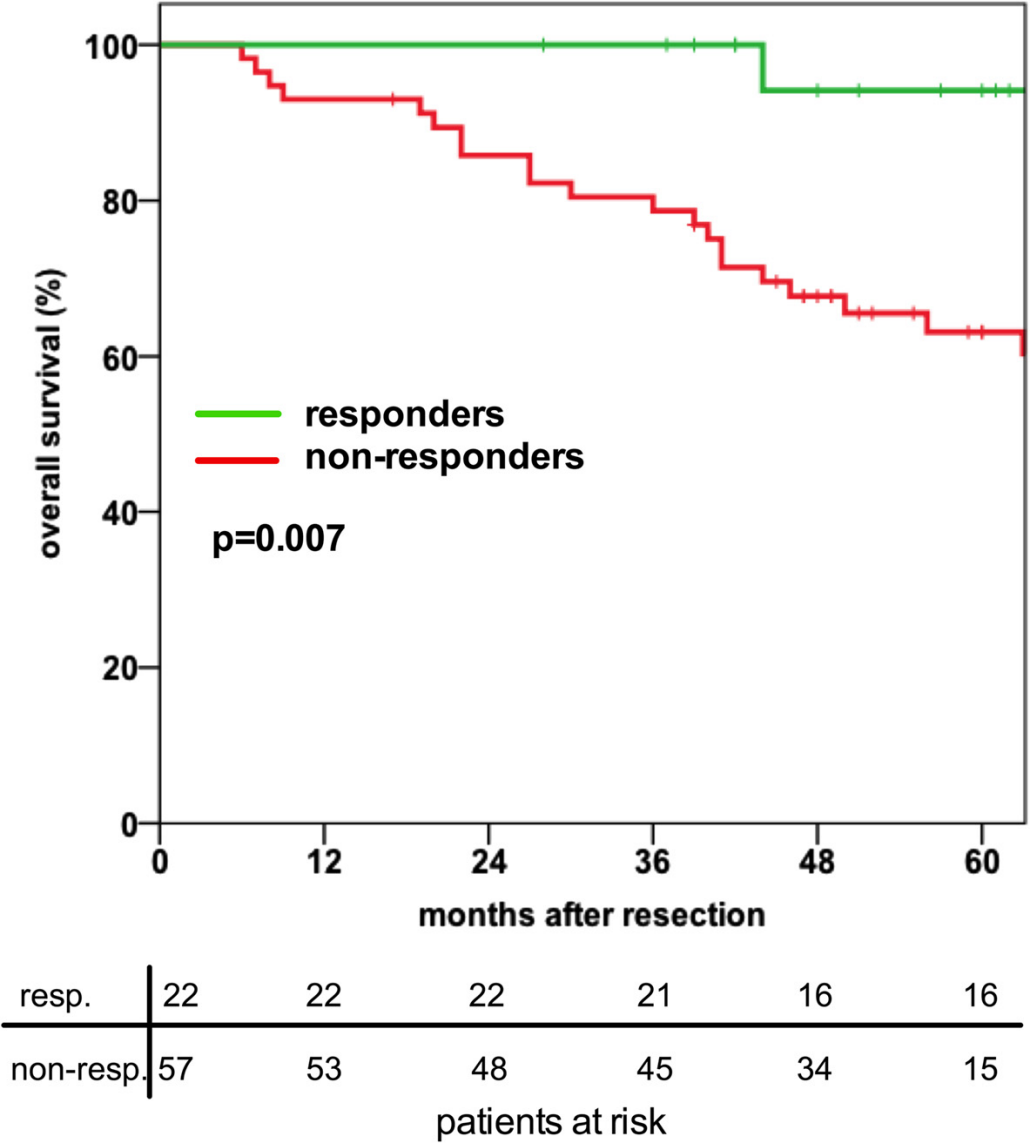
In the group of patients receiving neoadjuvant chemoradiation (*n* = 79) the overall survival of responders is significantly better compared to non-responders

All patients that received neoadjuvant chemoradiation were divided into histopathological responders (ypT0-T2 ypN0) and non-responders (ypT3-4 or ypN1). Histopathological response was observed in 22/79 patients (27.8 %). Two patients had a complete histopathological response (ypT0pN0) and in both patients no CTC were detected. Response to neoadjuvant chemoradiation has a major impact on long-term survival. This was evident by a significantly worse five-year overall survival rate for non-responders (60 %) compared to responders (95 %) (*p* = 0.007, log rank test) (Fig. 2). Response to neoadjuvant chemoradiation was independent of patient’s age, sex or tumor localisation in the rectum as tested with crosstab analysis.

Circulating tumor cells were detected in 54.4 % (31/57) of non-responders, whereas in responders circulating tumor cells were detected in 27.2 % (6/22) of the patients (*p* = 0.030 χ^2^-test, Table 3). The presence of circulating tumor cells after neoadjuvant chemoradiation was not influenced by tumor localisation. The incidence of circulating tumor cells was higher in patients with lymph node metastases (pN+) (55.8 % vs. 40 %), this not being statistically significant (*p* = 0.161 χ^2^-test, Table 3). Patients with UICC stage IV disease revealed the highest presence of circulating tumor cells after neoadjuvant chemoradiation (66.6 %), whereas only in 30.4 % of patients with UICC stage I had circulating tumor cells (Table 3).

**Table 3:** Detection of CK20 positive tumor cells (CTC) in the blood of patients with rectal cancer after neoadjuvant RCTX

Variable	Category	Rate of CK20 positive CTC	χ^2^-test (p-values)
RCTX	Responder	27.2 % (6/22)	*p* = 0.030
	Non-responder	54.4 % (31/57)	
Tumor localisation	Upper 1/3	54.5 % (6/11)	*p* = 0.848
	Middle 1/3	46.3 % (19/41)	
	Lower 1/3	44.4 % (12/27)	
pN- Status	pN+	55.8 % (19/34)	*p* = 0.161
	pN-	40 % (18/45)	
UICC Stage	I	30,4 % (7/23)	*p* = 0.265
	II	52.3 % (11/21)	
	III	51.7 % (15/29)	
	IV	66.6 % (4/6)	

## Discussion

According to current guidelines most patients with locally advanced rectal cancer are scheduled for neoadjuvant chemoradiation. The problem of local recurrence has been effectively reduced by this multimodal treatment. Even so, patients with advanced rectal cancer are still at high risk to develop distant metastases [16]. It is a widely accepted hypothesis, that dissemination of tumor cells from the primary tumor is a precondition for distant metastases and tumor recurrence. According to the “revisited” hypothesis of “seed and soil”, it does not only depend on the cell itself, but also on local environmental factors, whether circulating tumor cells can develop and grow out into liver and lung metastases [17]. Despite the inconsistency in the detection methods of CTC, the majority of studies published in the last years reported poor prognosis when CTCs are detected in colorectal cancer patients [7].

In addition to the prognostic value of CTC, the detection of CTC may be useful to serve as a predictive marker for therapy response. There is a wide spectrum of response to neoadjuvant chemoradiation, which creates an urgent necessity to predict the responders and non-responders in order to keep non-responders form unnecessary, potentially harmful treatment.

Only a few studies have evaluated the role of CTC in patients with locally advanced rectal cancer being candidates for neoadjuvant chemoradiation. Kienle et al. were the first to demonstrate in a small cohort of patients that RCTX leads to a clearance of CTC, which is associated with a decreased detection rate of CTC [18]. These results have been confirmed by other small monocentric trials [12, 19, 20], whereas only two studies have shown that CTC can be serve as marker for response after RCTX [12, 19]. We have shown in this so far largest series of patients that there is a correlation of response after neaodjuvant RCTX and detection of CTC.

In our study we used Ficoll extraction of MNC’s followed by CK20 RT-PCR to detect CTC. With an overall detection rate of 30 % this system is more sensitive than anti-EpCAM based binding capture technique (i.e., CellSearch™) which has recently been introduced. For rectal cancer patients detection rates of 19 % with the CellSearch™ system have been reported [19]. Recently, it has been shown that including CK20 as a biomarker for detection of CTC, the sensitivity of the CellSearch^TM^ system is efficiently enhanced [21], underlying the important role of CK20 in detecting CTC. Probably many CTC stay undetected due to an epithelial mesenchymal transition process (EMT) [22] of tumor cells or due to a CTC population with atypical characteristics which has been described earlier [23]. This group of CTC may prove to be very important for the treatment of the metastatic disease, as they represent stem cell-like cancer cells that most likely do not respond well to current therapeutic regimens.

In our series of patients with rectal cancer we were not able to show a correlation between CTC and tumor stage or overall survival. These results are contradictory to our own analysis of more than 500 colon cancer patients, demonstrating a negative correlation between detection of CTC and overall survival (data not shown). Most of the published series that have shown a negative prognostic impact of CTC (for overview see meta analysis [7]) do not differentiate between colon and rectal cancer patients. Our results are consistent with recent results from studies focusing only on rectal cancer patients, which were also not able to show a correlation between CTC and overall survival or tumor stage [12, 19, 20]. Even analyzing subgroups of our data (tumor localisation in the upper 1/3 of the rectum; only patients without neoadjuvant RCTX) we found no negative prognostic impact of CTC. Therefore, we have to conclude that tumor biology with regard to the impact of CTC is diverse between colon and rectal cancer. The blood drainage from the tumor might be an explanation for this different behavior. It has already been shown, that in patients with low rectal cancer the detection rate of CTC is higher in central venous blood than in the blood from the mesenteric vein compared to tumors in the middle and upper rectum [24]. In our series of patients the detection rate of CTC in the peripheral blood was not significantly different between tumors of the lower part compared to tumors in the middle and upper part of the rectum (data not shown).

The limitation of our study is, that we only measured CTC prior to surgery and not at different time points before and during neoadjuvant RCTX. Historically, that was not planned, because the focus was initially on patients with colon cancer, scheduled directly for the operation without any neoadjuvant treatment. We know from previous reports, that neoadjuvant RCTX is associated with a decreased detection rate of CTC [19]. We have shown a significantly decreased rate of CTC in responders after neoadjuvant RCTX. It would be very interesting, if this difference is already apparent before the treatment or at which time point after initiation of RCTX a significant difference in the detection rate can be detected. Goal for the future is to implement serial measurements of CTC before, during and after RCTX as a “liquid biopsy” to evaluate in which patients the CTC are effectively cleared from the blood during RCTX. Corresponding to PET-CT evaluation of patients with esophageal cancer at the beginning of neoadjuvant chemotherapy to guide the treatment [25], measurement of CTC during the beginning of neoadjuvant RCTX might be helpful to distinguish responders from non-responders and possibly preventing non-responders from potentially harming, ineffective RCTX.

## Conclusions

Our study supports the hypothesis of a correlation between the decrease in CTC and response to neoadjuvant RCTX for rectal cancer patients. For future investigations we suggest that a further improvement of the detection technique of CTC with a combinational approach of CK20 and EMT markers might be helpful to further elucidate the discrepancy between colon and rectal cancer biology concerning the prognostic relevance of CTC. In addition, detection of CTC should be incorporated in future studies in locally advanced rectal cancer with the aim to better select candidates for neoadjuvant RCTX.

## Additional file

Additional file 1:
**The figure shows a sample results of CK20 RT-PCR from 16 patients, including two positive controls (colon cancer cell line HT29).** (TIF 2824 kb)
